# Family participatory clown therapy in venipuncture in hospitalized children: A non-randomized controlled trial

**DOI:** 10.1371/journal.pone.0305101

**Published:** 2024-07-25

**Authors:** Tianyu Chen, Qiying Chen, Zhenhua Lin, Jingfang Ye

**Affiliations:** 1 Department of Nursing, Quanzhou Medical College, Quanzhou, Fujian, China; 2 Department of Pharmacy, The Second Affiliated Hospital of Fujian Medical University, Quanzhou, Fujian, China; Ain Shams University Faculty of Nursing, EGYPT

## Abstract

**Objective:**

To explore the effectiveness of family participatory clown therapy in venipuncture in hospitalized children.

**Methods:**

We recruited 104 children aged 3 to 6 years for a non-randomized controlled trial from March to December 2022. All participants required peripheral venepuncture infusions for treatment. The children were assigned to either the control group (n = 52) or the experimental group (n = 52).Standard care was utilized in the control group. In the experimental group, two clown nurses and a parent provided family participatory clown therapy for 35–45 minutes per child before, during, and after venipuncture. We assessed children’s pain (FLACC and W-B FPS), anxiety (VAS-A), medical fear (CFS), crying incidence, compliance, parental anxiety (S-AI), and parental satisfaction.

**Results:**

At venipuncture, the FLACC score was lower in the experimental group (4.46±2.053) compared to the control group (5.96±2.441), the W-B FPS score was also lower in the experimental group (4.96±2.392) than in the control group (6.35±2.266), with a statistically significant difference (*P*<0.05).The children in the experimental group had lower levels of anxiety, medical fear, crying, and parental anxiety than the control group. In addition, child compliance and parent satisfaction were higher in the experimental group than in the control group, with statistically significant differences (*P*<0.05).

**Conclusion:**

Family participatory clown therapy can reduce pain, anxiety, medical fear, and crying during venipuncture in children. It can also improve venipuncture compliance, reduce parental anxiety, and increase parental satisfaction.

## Introduction

Hospitalization is a stressor for children because of their unique developmental characteristics, such as their limited cognitive abilities, lack of self-control, fear of pain, and increased dependence on others, making hospitalized children more prone to pain, anxiety, and stress than others [1]. The preschool period is critical for children’s cognitive development and character formation. During this time, children are mentally immature, highly sensitive to pain, and prone to negative emotions such as nervousness, anxiety, and even fear of the disease itself and invasive medical procedures [2]. Negative emotions associated with invasive medical procedures can lead to problems in children, such as behavioral disorders, increased analgesic use, and general anxiety, which can disrupt normal diagnosis and treatment procedures [3]. Venipuncture is considered an invasive operation, adults can usually tolerate intravenous infusion, but children due to their young age and immaturity in physiology and intelligence, are prone to fear of pain, do not adapt to the unfamiliar environment of the hospital, have resistance to medical staff, have weak self-control, and cannot cooperate well with the work of medical staff, which makes venipuncture very difficult for children. In addition, repeated venipunctures failures will also cause children to have fear and resistance, thus affecting the effectiveness of therapy in children to a certain extent [4].

Children often describe needling-related operations as their most painful memory of treatment [5]. An analysis of a survey of children aged 3–17 years showed that children aged 3–6 years (36%) were more likely to experience moderate to severe pain during venipuncture than children aged 7–17 years (13%) [6]. In another study, 50% of children reported high levels of anxiety during venipuncture without pharmacologic or nonpharmacologic interventions. Young children had a higher level of anxiety (83%) than prepubescent children (7–12 years, 51%) and adolescents (12 years and older, 28%) [7, 8]. Memories of painful medical procedures may increase anxiety about subsequent medical procedures by influencing the child’s perception of pain [9]. Emotional changes caused by pain can suppress the body’s immune mechanisms and organ functions. This can be detrimental to the child’s physical and mental development, as well as the child’s recovery from the disease. Furthermore, painful stimuli can cause behavioral changes in children, such as loud crying and resistance. This can cause the needle to move away from the puncture site, leading to unwanted consequences such as tissue damage and additional bleeding from the needle [10]. Adverse medical experiences such as pain, fear, and anxiety in hospitalized children are inevitably transferred to their parents, which can cause stress for them and potentially trigger emotional disturbance [11]. Therefore, it is necessary to actively take effective measures to reduce the pain and anxiety associated with venipuncture in hospitalized children and to improve compliance to effectively ensure treatment outcomes.

In the recovery, daily care and prognosis of their child’s disease, parents play an important role. Family participatory care is a care model that involves the participation of family members in non-medical routine care, under the guidance of specialized nurses. The nurses train family members in relevant knowledge and instruct them on care methods [12]. Family participatory care encourages parents to participate in the daily care of their children. This not only improves the child’s compliance with medical procedures, but also increases parents’ knowledge of the disease and related caregiving skills, which is important for the treatment and recovery of a child’s disease [13]. Clown therapy is the application of clowning techniques derived from the circus to medical situations. It is a non-pharmacological intervention based on positive psychological such as humor, using comical clown figures, exaggerated character movements, and various behavioral techniques to induce positive emotions in patients and promote their recovery [14, 15]. Two systematic reviews and meta-analyses have suggested that clown therapy may be beneficial for pain relief when compared to standard care [16, 17]. Another systematic review and meta-analysis showed that clowning seems to lower children’s anxiety [18]. Several countries around the world have successfully used clown therapy in hospitals and communities. However, there have been few studies that have combined family participatory care with clown care in clinical settings. Therefore, in this study, we combined family participatory care with clown care in venipuncture of preschool (3-6years)hospitalized children with the hope to entertain them, distract them, relieve the pain, anxiety, and fear caused by venipuncture, and improve their compliance to some extent, so that they can move from passive cooperation to active cooperation and achieve better treatment outcomes. We also wanted to determine whether this model of care could reduce parental anxiety and increase parental satisfaction.

## Materials and methods

### Participants

From March 2022 to December 2022, the participants were recruited from the pediatric ward of a hospital in Quanzhou City, Fujian Province. The inclusion criteria for the study were as follows: children between the ages of 3 and 6 years who had been examined and evaluated by a physician, had normal hearing and vision, and did not exhibit any psychoneurological symptoms. Exclusion criteria were as follows: acute painful disease, critical disease, neurocognitive disorders, communication disorders, use of analgesics, concurrent participation in other studies. Inclusion criteria for children’s parents were: no history of mental illness, no communication impairment, and no cognitive impairment as assessed by medical evaluation; and the exclusion criteria was concurrent participation in other studies. Reference measurement data two-sample mean comparison sample content estimation formula to calculate the sample of this study, where α = 0.05, β = 0.1.The study’s primary endpoint was pain. According to the relevant literature [19], the pain score of the children in the test group was 3.92±2.41, while the control group had a pain score of 5.24±1.94. The sample size was calculated to be 47, and after accounting for a 10% rate of lost visits, the final sample size was determined to be 52 cases for each of the two groups. To prevent mutual influence, we utilized the convenience sampling method. The control group consisted of children admitted between March and July 2022, while the experimental group consisted of children admitted between August and December 2022. Each group included 52 cases. All children and parents who participated in the study were informed that participation was voluntary. They were also informed that participation or non-participation would in no way affect their right to treatment and care, and that they had the right to withdraw from the study at any time for any reason. This study adhered to the tenets of the Declaration of Helsinki. Registered with China Medical Research Registration and Filing Information System (NO.HSR-23-000192).This study was approved by the Ethics Committee of Quanzhou Medical College (NO.2021028)

### Standard intervention

Both groups utilized the same model of indwelling needle. Venipuncture was performed by members of the intravenous therapy team using standardized operating procedures. The control group children received standard care for related diseases after admission. This included medication care, diet care, life care, disease care, and health education. At the time of venipuncture, we verified the child’s basic information, helped them assume an appropriate position, and informed the child and parents about the purpose of the needle and related precautions. We answered the child’s and parent’s questions and provided verbal encouragement to the child during venipuncture. In the experimental group, family participatory clown therapy was implemented based on standard care, and the interventions are described below.

### Family participatory clown therap procedurey

#### Team members

There were seven team members: two for intervention implementation, two for data collection and analysis, and one for coordination and quality control during the intervention; in addition, there was a pediatrician and a nurse to assist the interventionist in implementing the intervention.

#### Team training

Experts were invited to provide training to the members over four sessions, each lasting 1.5 hours. The text covers the psychological characteristics of children, communication skills, the origin, purpose, theoretical basis, specific process, and research progress of clown therapy, the selection of clown images and props, performance and game skills, instruction on commonly used verbal phrases and exaggerated body movements, role-playing, participatory care for the family, and key points of cooperation for parental participation. After completing the training, an assessment must be passed before the intervention can take place.

#### Pre-intervention preparation

Research the cartoon characters, animated stories, games, and performances most popular with children. Prepare appropriate props such as costumes, dolls or masks, related improvisation, role-playing, magic, and game items, as well as animated videos related to the hospital theme.

#### Intervention programming

Through literature review, interviews, and group discussions, referring to the practical experience at home and abroad, and combining the characteristics of children’s psychological development and the actual situation in China, incorporating children’s favorite elements, we first formulated a localized family participatory clown care program. This program was then reviewed by experts and underwent pre-experimentation. To enhance the program, experts recommend including post-venipuncture interventions. Additionally, increasing parental involvement is suggested to help children feel more relaxed and willing to participate in the activities. The pre-experiment showed that some of the Western humor was not applicable, the lack of flexibility of the intervener, and the possibility that blowing bubbles could cause environmental hygiene problems and hospital-acquired infections. Taking into account the recommendations of the experts and the problems identified in the pre-experiment, the intervention program was revised after discussion among the team members.

#### Intervention program implementation

Assess the child’s age, preferences, emotional state, and cognitive development. Introduce and train the child’s parents in family participatory clown therapy to enable their full participation in the program. Based on the venipuncture procedure, the intervention was divided into three periods: before, during, and after venipuncture. Each period was performed by two clown nurses and a parent of the child. The intervention lasted for 35–45 minutes. Before the venipuncture: The clown nurses improvises according to the child’s preferences and plays games with the child and parents. They then simulate the venipuncture procedure and guide the parents and child to the venipuncture site by singing, dancing, and imitating an animal’s gait. During venipuncture: Gently touch the child’s hand and place a favorite cartoon sticker on the child’s arm or forehead while providing verbal encouragement. Make an agreement with the child that a surprise gift will be given if the venipuncture is done well. An animated hospital-themed video was shown and the child was communicated with within the context of the video’s storyline, then the clown nurse and parent simulated venipuncture, providing some verbal cues and encourageme. After venipuncture: Continue to interact with the child through performing, playing, and storytelling. The child was praised again for his brave behavior and given a pre-agreed gift as a reward. Discuss with the children and parents their feedback on participating in the program and gather suggestions for improvement. The family participatory clown therapy intervention program is presented in S1 Table.

### Primary outcome measures

#### Pain

The FLACC scale [20] was used to objectively assess the children’s pain response, including face, legs, activity, cry, consolability. Each item was scored as 0, 1, or 2, with a total score of 0–10, with a higher the score indicated a more painful experience. The Wong-Baker Face Pain Scale (W-B FPS) [21] was used to subjectively assess pain experienced by the child. The W-B FPS consisted of six facial expressions ranging from smiling to calm to crying on a scale of 0 to 10. Children were asked to indicate which expression best represented their pain level during venipuncture, with higher scores indicating more pain.

### Secondary outcome measures

#### Anxiety

The Visual Analogue Scale for Anxiety (VAS-A) is a 10 cm line, with the leftmost (0 cm) portion being “calm, no anxiety” and the rightmost (10 cm) portion being “very anxious”. Higher scores indicate greater anxiety [22]. The VAS-A is designed for children six years and older who can understand and complete the task. A study found a correlation between parents’ predictions of their child’s reactions and the child’s behavior and self-report [7]. The only significant predictor of pain and distress in 3–6 year olds was the parent’s prediction, which was highly correlated with all measures of distress [7]. The VAS-A has also been used for parental assessment of anxiety in children aged 2–10 years old [23]. Therefore, in this study, the child’s anxiety level was reported by the parents, who marked a spot on the line to indicate it.

#### Medical fear

The Children’s Fear Scale (CFS) was used to evaluate the children’s medical fears. McMurtry et al. (2011) developed the CFS to evaluate children’s fear of undergoing a painful medical procedure [24]. Interrater reliability (Time 1: rs = 0.51, p < 0.001) and test-retest reliability (rs = 0.76, p <0 .001) of the CFS for measuring children’s fear during venipuncture were supported [24]. The CFS has been previously used to assess fear in children aged 3 to 12 years [25, 26]. It is reliable and valid. The CFS uses a five-face scale to measure fear levels, ranging from a neutral expression (0 = no fear) to an extreme fearful expression (4 = extreme fear). The child is presented with these faces and asked to select the one that best represents their current level of fear.

#### Crying

The study observed the children for crying both during and one minute after venipuncture.

#### Venipuncture success rate

The one-time success rate of venipuncture was observed.

#### Compliance

Compliance was categorized as three types. Poor compliance indicated children who were strongly resistant and unable to cooperate after intervention, persuasion, and reassurance, requiring assistance with restraint for venipuncture. General compliance indicated children who were mildly resistant but could cooperate after persuasion and reassurance. Good compliance indicated complete and active cooperation without assistance.

#### Anxiety of the child’s parent

The State Anxiety Inventory (S-AI) developed by Spielberger et al. [27] was used to assess anxiety in stressful situations; it was designed to reflect immediate or recent experiences or feelings of fear, tension, apprehension, and nervousness at a specific time. The reliability and validity of the Chinese version of the S-AI are good, with a reliability coefficient of 0.9062 and an internal consistency coefficient of 0.894 [28, 29]. The scale consists of 20 questions and with scores ranging from 1 to 4 (1 = not at all, 2 = some, 3 = moderate, 4 = very pronounced; i.e., positive emotions are scored in reverse order), where higher scores indicated a higher anxiety level. The child’s parent selected the appropriate level of anxiety based on their own experience.

#### Satisfaction of child’s parents

Parental satisfaction was evaluated by asking the following question: “How satisfied are you with the overall performance of venipuncture?” Parental satisfaction was evaluated on a Likert scale of ranging from 1 to 5 (1 = unsatisfied, 2 = relatively unsatisfied, 3 = generally satisfied, 4 = relatively satisfied, 5 = very satisfied). This index was collected 10 min after venipuncture completion [30].

#### Statistical analysis methods

Statistical analysis was performed on the data using SPSS 21.0 software. The measurement data were presented as mean ± standard deviation. For data meeting parametric assumptions, two-sample independent t-tests were used for between-group comparisons and paired t-tests for within-group comparisons. In the case of non-parametric assumptions, the independent Mann-Whitney test was used for between-group comparisons and the paired Wilcoxon test for within-group comparisons. Count data were expressed as frequencies and percentages, and the Chi-squared test was used to compare between groups. Repeated-measures analysis of variance (ANOVA) was used to analyze the data collected at multiple time points. The p-values were adjusted using the false discovery rate.

## Results

### Basic characteristics of children and their parent in two groups

This study included a total of 104 cases, with 52 in both the experimental and control groups. No shedding occurred during the study. The flow of participants through each phase of the study is presented in S1 Fig. Each child was accompanied by a parent. The children in the experimental group were (4.67±0.944) years old, Ten had a history of venipuncture and their parent were (34.04 ± 5.448) years old, while the children in the control group were (4.75 ± 0.860) years old and 13 had a history of venipuncture and their parent were (33.73 ± 5.318) years old, with no statistically significant difference between the two groups (*P* > 0.05). There was also no statistically significant difference in the gender of the children and the gender and education of the parents in the two groups (*P*>0.05), as shown in Table 1.

**Table 1:** Basic characteristics of children and their parent.

Characteristic	Control group (n = 52)	Experimental group (n = 52)	P-value
Gender			
Boy, n (%)	28 (53.85)	31 (59.62)	0.553^a^
Girl, n (%)	24 (46.15)	21 (40.38)	
Age (mean±SD/years)	4.75±0.860	4.67±0.944	0.730^b^
History of venipuncture			
yes, n (%)	13 (25.00)	10 (19.23)	0.478^a^
no, n (%)	39 (75.00)	42 (80.77)	
Gender of the child’s parent			
Male, n (%)	16 (30.77)	14 (26.92)	0.665^a^
Female, n (%)	36 (69.23)	38 (73.08)	
Age of the child’s parent (mean±SD/years)	33.73±5.318	34.04±5.448	0.771^c^
Education of the child’s parent			
Junior high school and below, n (%)	5 (9.61)	4 (7.69)	0.799^a^
High school/secondary school, n (%)	15 (28.85)	18 (34.62)	
College and above, n (%)	32 (61.54)	30 (57.69)	

^a^ Chi-square test

^b^ Independent samples Mann-Whitney test

^c^ Independent samples t-test.

### Comparison of pain levels between the two groups

The FLACC score at venipuncture was lower in the experimental group (4.46±2.053) than in the control group (5.96±2.441), with a statistically significant difference (*FDR* = 0.002). The W-B FPS score at the time of venipuncture was lower in the experimental group (4.96±2.392) than in the control group (6.35±2.266), with a statistically significant difference (*FDR* = 0.008), as shown in Table 2.

**Table 2:** Multiple comparisons of FDR for pain levels between the two groups.

Items	Control group (n = 52)	Experimental group (n = 52)	FDR
FLACC(mean±SD)	5.96±2.441	4.46±2.053	0.002
W-B FPS(mean±SD)	6.35±2.266	4.96±2.392	0.008

### Comparison of anxiety levels between the two groups

The difference in anxiety scores between the two groups of children before the intervention was not statistically significant (*FDR* = 0.611). The anxiety score during venipuncture was lower in the experimental group (3.98±1.213) than in the control group (4.75±1.507), and the difference was statistically significant (*FDR* = 0.008), as shown in Table 3.

**Table 3:** Multiple comparisons of FDR for anxiety levels between the two groups.

	Control group (n = 52)	Experimental group (n = 52)	FDR
Pre-intervention	2.83±0.834	2.69±0.981	0.611
During venipuncture	4.75±1.507	3.98±1.213	0.008
FDR	0.000	0.000	

### Comparison of medical fear levels between the two groups

The difference in medical fear scores between the two groups of children before the intervention was not statistically significant (*P* = 0.434). The medical fear score was lower in the experimental group (2.31±0.755) than in the control group (3.54±0.699) at the time of venipuncture, and the difference was statistically significant (*P*<0.001). The medical fear score was lower in the experimental group (0.56 ± 0.608) than in the control group (1.44 ± 0.639) 10 min after venipuncture, and the difference was statistically significant (*P*<0.001),as shown in Table 4.

**Table 4:** Comparison of repeated ANOVA for medical fear levels in two groups.

	Control group (n = 52)	Experimental group (n = 52)	P-value
Pre-intervention	3.38±0.796	3.50±0.700	0.434
During venipuncture	3.54±0.699	2.31±0.755	0.000
10 minutes after venipuncture	1.44±0.639	0.56±0.608	0.000
P-value	0.000	0.000	

### Comparison of crying, compliance, and parent satisfaction between the two groups

The incidence of crying during venipuncture was lower in the experimental group (40.38%) than in the control group (88.46%), and the difference was statistically significant (*FDR*<0.001). The rate of children stopping crying within 1 minute was higher in the experimental group (57.14%) than in the control group (47.83%), but the difference was not statistically significant (*FDR* = 0.611). The success rate of primary venipuncture was better in the experimental group (94.23%) than in the control group (90.38%), but the difference was not statistically significant (*FDR* = 0.713). The compliance rate of children in the experimental group (90.38%) was higher than that of the control group (80.77%), and the difference was statistically significant (*FDR* = 0.025). The satisfaction rate of parents of children in the experimental group (4.71±0.572) was higher than that of the control group (4.13±0.908), and the difference was statistically significant (*FDR* = 0.001), as shown in Table 5.

**Table 5:** Multiple comparisons of FDR for crying, compliance, and parent satisfaction between the two groups.

Items	Control group (n = 52)	Experimental group (n = 52)	FDR
crying			
yes, n (%)	46 (88.46)	21 (40.38)	0.000
no, n (%)	6 (11.54)	31 (59.62)	
Stop crying within 1 minute			
yes, n (%)	22 (47.83)	12 (57.14)	0.611
no, n (%)	24 (52.17)	9 (42.86)	
The success of one venipuncture			
yes, n (%)	47 (90.38)	49 (94.23)	0.713
no, n (%)	5 (9.62)	3 (5.77)	
compliance			
Poor compliance,n (%)	10 (19.23)	5 (9.62)	0.025
General compliance,n (%)	31 (59.62)	25 (48.08)	
Good compliance,n (%)	11 (21.15)	22 (42.31)	
Satisfaction (mean±SD)	4.13±0.908	4.71±0.572	0.001

### Comparison of parental anxiety levels between the two groups

The difference between the anxiety scores of the parents of the children in the two groups before the intervention was not statistically significant (*FDR* = 0.654). After the intervention, the anxiety scores of the experimental group (35.54±10.573) were lower than those of the control group (40.10±10.700), and the difference was statistically significant (*FDR* = 0.011). The experimental group showed a statistically significant decrease (*FDR*<0.001) in parental anxiety scores during the post-intervention period compared to the pre-intervention period, as shown in Table 6.

**Table 6:** Multiple comparisons of FDR for parental anxiety levels between the two groups.

	Control group (n = 52)	Experimental group (n = 52)	FDR
Pre-intervention	38.52±11.344	37.71±11.157	0.654
Post-intervention	40.10±10.700	35.54±10.573	0.011
FDR	0.000	0.000	

## Discussion

### Family participatory clown therapy relieved pain during venipuncture in children

Pain caused by invasive operations is prevalent during treatment in children. These children often resist treatment because of pain. Pain can not only have short-term effects on children, but it can also negatively affect their future healthcare behaviors. Therefore, effective pain care for children based on their psychological characteristics and cognitive development is essential [19]. The results of this study showed that the subjective and objective pain scores of children in the experimental group were lower than those in the control group, and the difference was statistically significant (*P*<0.05), indicating that family participatory clown therapy could alleviate the pain level of venipuncture in children, which is consistent with the findings of Ding et al. and Kurudirek et al. [31, 32]. Effective non-pharmacological interventions can help reduce pain perception in children and have the advantages of simplicity and ease of use, low cost, safety, and fewer adverse effects compared to pharmacological interventions [33]. As a non-pharmacological intervention method, clown therapy is based on positive psychology [34], which integrates contextual, behavioral, and psychological approaches, according to the child’s age, physical condition, emotional state, personality characteristics, interests, and preferences. With the help of exaggerated and humorous dances and postures, animated audio and video, funny music, and other tools, we can create a relaxing atmosphere, soothe the child’s emotions, and divert the child’s attention from venipuncture pain. The attention span of children is limited, and the resources of their brain’s information processing system are occupied during pleasant distracting activities, which reduces an individual’s concentration on pain, increases their tolerance to pain, relieves the intensity of pain perception, and also facilitates the smooth implementation of therapeutic care operations [15]. At the same time, the involvement of the child’s parents throughout the process helps the child feel more secure in the face of a medical operation, making them more willing to communicate with the clown nurse.

In this study, the intervention process was based on animated characters of interest to the child through localized clown images, and the interventionist used exaggerated body language and expressions with the child’s parents to cooperate with the child’s interaction, fully mobilizing the child’s positive emotions, making the child happy, and reducing the perception of pain [35]. On the other hand, the pleasant experience of clown therapy, such as humor and “laughing,” can eliminate the negative emotions of the child and generate positive emotions, stimulating the cerebral cortex (i.e., the nucleus accumbens, amygdala, frontal and temporal lobe area) to produce more dopamine and endorphins, which can help reduce the pain of venipuncture in children [36].

### Family participatory clown therapy relieved anxiety during venipuncture in children

Because of their immature physical and mental development, children have limited cognitive ability and low self-control, and are prone to anxiety during medical treatment [37]. In addition, when the child enters the hospital, they are far from their familiar family and friends, and have to face the monotonous hospital environment, the unfamiliar people around them, and the medical staff, which will naturally lead to anxiety. These emotional and stressful reactions can cause alterations in central nervous system function and have adverse effects on the affected child [38]. Therefore, effective measures to alleviate anxiety in children are of increasing concern to pediatric nursing staff.

The results of this study showed that the anxiety scores of the children in the experimental group were lower than those of the control group, and the difference was statistically significant (*P*<0.05), indicating that family participatory clown therapy could reduce the anxiety of the children, which is consistent with the findings of Lopes-Júnior et al. [39]. In clown therapy, the interventionist interacts with the child before the venipuncture in collaboration with the child’s parents, attracting the child’s attention through humor, childlike dressing, exaggerated body language, expressions, and flexible performances, which can bring joy to the child and help them relax, thus stimulating their positive emotions and other mechanisms, and relieving their anxiety [40, 41]. During the interaction with the child, they can continue to watch their favorite cartoons and play familiar games; this establishes a safe and relaxing environment for the child, which also helps them release their emotions and overcome their anxiety. During this process, the child will gradually become familiar with the environment. This will also make the child feel safe and relaxed, and reduce fear and anxiety about the unfamiliar environment during hospitalization. Parental companionship is crucial to treatment and a source of security for children, as it plays a vital role in adapting to life in the hospital and overcoming medical fears and anxiety. In the process of family participatory clown therapy, parents give active attention to the child during play and provide timely encouragement and guidance, which plays an important role in supporting the child’s sense of security and overcoming anxiety.

### Family participatory clown therapy alleviated medical fears

Medical fear is a common psychological reaction in hospitalized children, which can reduce their compliance with medical care and even cause psychological trauma and affect rehabilitation. Therefore, it is necessary to intervene in hospitalized children and to assist them in regulating their medical fears for their treatment and recovery process. This study showed that children in the experimental group had lower medical fear scores than the control group. The difference was statistically significant (*P*<0.05), indicating that family participatory clown therapy could alleviate medical fears. Medical fear in children has multifaceted causes. Ganga et al. [42] concluded that medical fear in children mainly arises due to unfamiliar hospital environments, painful injection treatment, and contact with strangers. When children are hospitalized, they must leave their familiar living environment and enter an unfamiliar hospital environment, and they often develop fear. During hospitalization, various medical operations such as venipuncture, injections, and blood collection can produce painful stimuli. A large part of the child’s medical fear is due to fear of pain, and different experiences of pain can influence the child’s medical fear [43]. Family participatory clown therapy can make children familiar with the environment and relieve the pain of venipuncture by interacting with them, thus relieving their medical fears.

### Effect of family participatory clown therapy on venipuncture compliance, crying incidence, and success rate of primary venipuncture in children

The results of this study showed that the compliance of venipuncture in children in the experimental group was higher than that in the control group, and the incidence of crying in children in the experimental group was lower than that in the control group, with statistically significant differences (*P*<0.05), suggesting that family participatory clown therapy helps to improve the compliance of venipuncture in children, reduce the incidence of crying in children, and make the process of venipuncture more pleasant. The results were similar to those of Yildirim et al. [44]. Due to their characteristics, children are prone to anxiety and fear because of the painful stimulation of venipuncture and the unfamiliar environment and strangers. This negative emotion will aggravate the child’s pain. Crying is the way children release emotional pressure, but crying and resistance will hinder the medical staff’s operation [45]. If coercion is used, although venipuncture can be done quickly, it can cause intense stress in the child. This is detrimental to the child’s physical and psychological development, as well as to subsequent medical operations. In this study, the interventionist was able to flexibly use everything available in the surrounding environment to perform according to factors such as the child’s age, personality, temperament, physical condition, cognitive development, interests, and emotional state. By interacting with the child during venipuncture, the interventionist reduced rejection and fear of the surrounding environment, distracted the child’s attention, reduced the child’s focus on pain, alleviated fear and tension about venipuncture, moved the child from passive acceptance to active cooperation, improved venipuncture compliance, reduced the occurrence of crying, and made the venipuncture experience more relaxing [10].

From the perspective of education and behavior, preschool children are in the stage of curiosity, hyperactivity, and easy imitation. As the first teachers of children, parents are the most trusted and dependable people for children, and their behavior and demeanor can implicitly influence the way children handle events [46]. In this study, the interventionist and the child’s parents simulated the procedure in front of the child during the child’s venipuncture. This was done in exaggerated and comical ways so that the child established a positive perception of the venipuncture procedure through their parents’ behavior and reactions, thus improving compliance [35]. At the same time, scenarios and games that simulate venipuncture allow for a more visual depiction of the specific venipuncture procedure. Thus, the child can understand it more easily, improving cooperation and compliance. On the other hand, the process of formulating an agreement, expressing praise, reaching an agreement, and formulating the appointment again in this study can effectively motivate the child, improve venipuncture compliance, and reduce crying [36].Some studies have shown that clown therapy shortens crying time [37]. However, the results of this study showed that there was no statistically significant difference in the rate of cessation of crying within one minute and the success rate of one venipuncture between the two groups. The reasons for this result may be related to the skilled puncture technique of the operator, the high success rate, and the fact that the parents took the child out of the stressful environment after the venipuncture.

### Family participatory clown therapy reduced anxiety and increased satisfaction for parent

The results of this study showed that the scores of S-AI of the parents of the children in the experimental group were lower than those of the control group, and the satisfaction scores of the parents of the children in the experimental group were higher than those of the control group, with statistically significant differences (*P*<0.05), indicating that family participatory clown therapy can reduce anxiety and improve satisfaction in parents of the children during venipuncture, which is similar to the findings of Sridharan et al. [47] and Mortamet et al. [48]. When children seek medical treatment, parents may show excessive anxiety and stress due to a lack of knowledge related to the disease and inexperience in care [37]. At the same time, parents of children with long-term hospitalization are constantly under significant psychological pressure and prone to anxiety and fear. The child’s mood also directly affects the parents’ mood fluctuations, including the child’s discomfort and fear of painful stimuli that manifest as crying and resistant behavior. This reaction often causes psychological stress and anxiety for the parents [49].

The traditional behavioral model of medical personnel tends to cause negative experiences in children, such as the “white coat phenomenon” or the “syringe phenomenon,” whereas family participatory clown therapy emphasizes the involvement of the child’s parents in the playful interaction between the interventionist and the child. Child-centered targeted games and videos are given according to the different characteristics and preferences of the child, and the child is amused with humor and fun to distract them, reduce their fear of venipuncture and the surrounding environment, reduce the perception of pain, reduce crying, promote positive emotions, and improve compliance and cooperation with treatment and care. Furthermore, the child’s parents can feel care and support in the process, which helps to reduce their psychological pressure to a certain extent, relaxes them, and reduces anxiety, thus increasing satisfaction [35]. At the same time, family participatory clown therapy can explain professional medical knowledge in a lifelike way, which also facilitates communication between the parents of the child and professional medical staff or clown nurses; it also increases the parents’ understanding of disease treatment and care, which can also temporarily relieve their psychological stress, reduce anxiety, and improve satisfaction with nursing care to a certain extent [50]. Family participatory clown therapy embodies the concept of “person-centered” care, which indirectly reduces doctor–patient disputes and lays the foundation for a good doctor–patient relationship [10]. Relevant studies have also shown that clown therapy not only benefits patients but also fosters an atmosphere of interaction between hospital staff and patients. This leads to more doctor–patient harmony, in which laughter and humor self-perpetuate [15].

### Study limitation

Because of the children’s age and cognitive development, their anxiety levels were assessed through their parents’ reports. While this method can reflect the children’s anxiety to some extent, it may not fully capture their true level of anxiety. Future studies should select more appropriate instruments for assessment based on the children’s level of cognitive development. Future studies could increase the sample size and conduct a multicenter study, as the current sample size is small. The intervention is composite. It includes not only the intervention of the clown nurse, but also stroking, colored stickers, encouragement, and animated videos. Therefore, it is difficult to determine the net effect of clown therapy.

## Conclusions

Family participatory clown therapy combines the family participatory care model with clown care through localized clown images, which can effectively reduce pain, anxiety, and medical fear of hospitalized children during venipuncture, reduce crying, improve compliance, relieve parents’ anxiety, and increase parents’ satisfaction with nursing operations. As a non-pharmacological intervention, clown therapy has been widely used in medical operations for pediatric patients abroad. However, clown therapy is still in its infancy in China, and there are few relevant scientific studies. It is suggested that, in the future, we can take into account the national conditions and cultural characteristics of China, optimize the intervention program, broaden the application field of clown therapy, explore its application to different medical operations, and further prove the effectiveness and sustainability of family participatory clown therapy.

## Supporting information

S1 Table Family participatory clown therapy intervention program. (DOC)

S2 Table TREND statement checklist. (PDF)

S3 Table Trial protocol. (DOC)

S1 Fig Flow of participants through each phase of the study. (DOC)
