# Supplementary material for: Family participatory clown therapy in venipuncture in hospitalized children: A non-randomized controlled trial
Source: PLoS One. 2024 Jul 25;19(7):e0305101. doi: 10.1371/journal.pone.0305101 (PMC11271897; doi:10.1371/journal.pone.0305101)
Supplement: S1 Table — (DOC) [file pone.0305101.s001.doc]

**S1 Table. Family participatory clown therapy intervention program**

| **Intervention time** | **Intervention method** | **Intervention measures** |
| --- | --- | --- |
| **Before venipuncture**  **（20 minutes）** | Parent training, role-playing, atmosphere creation, improvisation, games, mimicking the piercing process | ①The intervenor begins by communicating with the child and their parents to understand the child's age and hobbies, assess their emotional state and cognitive development, and introduce and train the parents on family participatory clown therapy. This includes explaining the source and purpose of clown care, the process and content of parental involvement, performance skills, and key points of cooperation.The interventionist asked the child about their favorite cartoon character and then dressed in the corresponding costume or held the corresponding doll to communicate with the child. The intervenor performed relevant improvisation based on the child's preferences to establish a trusting relationship.②Intervenor engage children and their parents in various activities such as balloon blowing, clay modeling, painting, origami, puzzles, doll play, storytelling, and magic tricks. Children are given the freedom to choose their preferred dolls, props, and balloon shapes.Through games, we can create a relaxed and joyful atmosphere to reduce the children's unfamiliarity with their surroundings. This will reduce their fear of medical treatment.③The intervenor simulated a medical operation situation with the children and their parents to reduce the children's fear of medical operations.For example,a toy needle or a long balloon can be used as a needle to simulate venipuncture with the child and their parents. This procedure aims to familiarize the child with venipuncture, reducing their fear of needles. Finally, the intervenor guides the parents and child to the venipuncture site using playful techniques such as singing, dancing, and imitating animal movements. |
| **During venipuncture**  **(10-15 minutes)** | Stickers, agreements, cartoons, music, dolls, role-play, improvisation, and praise | ①Before disinfecting the skin, the intervenor gently stroked the child's hand, comforted the child, and verbally encouraged the child by placing the child's favorite cartoon sticker on the child's arm or forehead. There is an agreement with the child that if the venipuncture is done well, there will be a mystery reward at the end of the procedure.②During venipuncture, create a comfortable environment for the child. This can be achieved by playing the child's favorite cartoons or music related to the hospital theme. Additionally, allowing the child to hold a favorite doll can provide a sense of security. The intervener and parents communicate with the child during the video storyline, imitating the venipuncture process and providing verbal cues to distract and relieve the child's tension. For example, they may say 'Ultraman can send energy to your body through this tube so that we can defeat the monsters.'During venipuncture, it is important to pay attention to timing and provide encouragement and praise to children to make them feel comfortable. |
| **After venipuncture**  **(5-10 minutes)** | Praise, rewards, improvisation, developing protocols, discussing feelings | ①Continue to interact with the child through performing, playing, and storytelling.②The child and parents were escorted to the ward. During this time, the child was commended for their bravery and given a pre-agreed gift as a reward for their behavior during the venipuncture③Develop a protocol with the child for the next venipuncture.④Discuss with the children and parents their feedback on participating in the program and gather suggestions for improvement. |
