# Supplementary material for: Family participatory clown therapy in venipuncture in hospitalized children: A non-randomized controlled trial
Source: PLoS One. 2024 Jul 25;19(7):e0305101. doi: 10.1371/journal.pone.0305101 (PMC11271897; doi:10.1371/journal.pone.0305101)
Supplement: S3 Table — (DOC) [file pone.0305101.s003.doc]

**Trial Protocol**

| **Project Title** | Family participatory clown therapy in venipuncture in hospitalized children: A non-randomized controlled trial | |
| --- | --- | --- |
| **Keywords** | Family participatory care; Clown therapy; Hospitalized children; Venipuncture | |
| **Type of Study** | B | A. Basic Research B. Applied Research C. Synthesis Research |
| **Summary of the main content of the study and expected research results**  Among the various ways of drug delivery, intravenous venipuncture occupies an important position, which is usually tolerated by adults, but children can't cooperate well due to their weak self-control, causing great difficulty in venipuncture, and repeated failure of venipuncture will also cause terror and resistance, which affects the therapeutic effect of the children to a certain extent. Therefore, the present study proposes the family participatory clown therapy as an intervention to reduce the pain of the children, improve compliance, ensure the therapeutic effect, and promote the recovery of the children. | | |

| **1、Scientific basis and significance of this study project**  Hospitalization is a stressor for children because of their unique developmental characteristics, such as their limited cognitive abilities, lack of self-control, fear of pain, and increased dependence on others, making hospitalized children more prone to pain, anxiety, and stress than others (1). In addition, children often experience varying degrees of anxiety or pain during treatments or invasive operations (2). Anxiety associated with invasive medical procedures can lead to problems in children, such as behavioral disorders, increased analgesic use, and general anxiety, which can disrupt normal diagnosis and treatment procedures (3). Venipuncture is considered an invasive operation; adults can typically tolerate intravenous infusion, but children— due to their young age and immaturity in physiology and intelligence—are prone to fear of pain, do not adapt to the unfamiliar environment of the hospital, have resistance to medical staff, have weak self-control, and cannot cooperate well with the work of medical staff, which makes venipuncture very difficult for children. In addition, repeated puncture failures will also cause children to have fear and resistance, thus affecting the effectiveness of therapy in children to a certain extent (4).  Children often describe needling-related operations as their most painful memory of treatment (5). Memories of painful medical procedures can increase anxiety about subsequent healthcare procedures by influencing the child’s perception of pain (6). In addition, emotional changes caused by pain can suppress the body’s immune mechanisms and organ functions, which is detrimental to the child’s physical and mental development, as well as their disease recovery. Furthermore, painful stimuli can cause behavioral changes in children, such as loud crying and resistance, which can deviate from the puncture site and cause undesirable consequences such as tissue damage and additional bleeding from the needle (7). In addition, adverse medical experiences such as pain, fear, and anxiety in hospitalized children are inevitably transferred to their parents, which can cause stress for them and potentially trigger emotional disturbance (8). Therefore, it is necessary to actively take effective measures to alleviate the pain and anxiety of venipuncture in hospitalized children and to enhance compliance, thus effectively ensuring treatment outcomes.  Parents play an important role in the recovery, daily care, and prognosis of their child’s illness. Family participatory care encourages parents to participate in the daily care of their children; this not only improves compliance with medical procedures but also enhances parents’ knowledge of the disease and related nursing skills, which is important for the treatment and recovery of a child’s disease (9). Clown therapy is the application of clowning techniques derived from the circus to disease situations; it is a non-pharmacological intervention based on positive psychological theories such as humor, using comical clown figures, exaggerated character movements, and various behavioral techniques to induce positive emotions in patients and promote their recovery (10,11).Several countries around the world have successfully used clown therapy in hospitals and communities. However, there have been few studies that have combined family participatory care with clown care in clinical settings.Therefore, in this study, we combined family participatory care with clown care in venipuncture of hospitalized children with the hope to entertain them, distract them, relieve the pain, anxiety, and fear caused by venipuncture, and improve their compliance to a certain extent, so that they can shift from passive cooperation to active cooperation and achieve better treatment results. We also aimed to determine whether this care model can alleviate parents’ anxiety and increase satisfaction.  **REFERENCES**  (1)Li HC. Evaluating the effectiveness of preoperative interventions: the appropriateness of using the Children’s Emotional Manifestation Scale. J Clin Nurs. 2007;16(10), 1919-1926. doi: 10.1111/j.1365-2702.2007.01784.x.China.  (2)Yu XY, Liu Y,Huang Y. Effects of clown care on children’s anxiety and pain: a meta-analysis. J Nurs. 2022;29(4), 21-26. doi: 10.16460/j.issn1008-9969.2022.04.021. China.  (3)Vagnoli L, Caprilli S, Messeri A. Parental presence, clowns or sedative premedication to treat preoperative anxiety in children: what could be the most promising option? Paediatr Anaesth. 2010 Oct; 20(10), 937-943. doi: 10.1111/j.1460-9592.2010.03403.x  (4)Liu J, Liu H. Discussion on application effect of psychological nursing in infantile intravenous infusion. Shanxi Med J. 2020;49(7), 914-915. doi: 10.3969/j.issn.0253-9926.2020.07.062. China.  (5)Wilson ME, Megel ME, Enenbach L,Carlson KL. The voices of children: stories about hospitalization. J Pediatr Health Care  2010 Mar-Apr; 24(2), 95-102. doi: 10.1016/j.pedhc.2009.02.008  (6)Crocker PJ, Higginbotham E, King BT,Taylor D, Milling TJ. Comprehensive pain management protocol reduces children’s memory of pain at discharge from the pediatric ED. Am J Emerg Med. 2012 Jul;30(6), 861-871. doi: 10.1016/j.ajem.2011.05.030  (7)Li JL. Study on the application of clown care in children’s vaccination. [dissertation]. [Shanxi]: Shanxi Medical University. 2020. doi: 10.27288/d.cnki.gsxyu.2020.000996.China.  (8)Yip P, Middleton P, Cyna AM, Carlyle AV. Non-pharmacological interventions for assisting the induction of anesthesia in children. Cochrane Database Syst Rev . 2009 Jul; 8(3), CD006447. doi: 10.1002/14651858.CD006447.pub2  (9)Jiang YQ, Wang ZL, Xiang MQ. Effects of family participation in nursing care in premature infants in neonatal intensive care units. Chin J Mod Nurs. 2021;27(5), 669-672. doi: 10.3760/cma.j.cn115682-20200325-02165 .China.  (10)Kurudirek F, Arıkan D. Effects of therapeutic clowning on pain and anxiety during intrathecal chemotherapy in Turkey. J Pediatr Nurs. 2020 Jul-Aug;53, e6-e13. doi: 10.1016/j.pedn.2020.01.015  (11)Ding YX, Li JL, Guo WJ, Sun LB, Chen C,Wang BQ. On the feasibility of introducing clown care to China. J Nurs Sci. 2019;34(21), 99-103. doi: 10.3870/j.issn.1001-4152.2019.21.099.China.  (12)Li, J.L., Zhang, P.L., Ding, Y.X., et al. (2020). Application of clown care in vaccination of preschool children. Chin. Nurs. Res. 34(10), 1811-1815. doi: 10.12102/j.issn.1009-6493.2020.10.028  **2.Research content**  **2.1research population**  **2.1.1 Sample Sources**  104 children admitted to the pediatrics department of a hospital in Quanzhou City from March 2022 to December 2022 who required venipuncture for infusion treatment.  **2.1.2 Inclusion criterias**  (1) The inclusion criteria of the children: children between the ages of 3 and 6 years who had been examined and evaluated by a physician, had normal hearing and vision, and did not exhibit any psychoneurological symptoms.  (2)The inclusion criteria for parents of the children: no history of mental illness, no communication impairment, and no cognitive impairment as assessed by medical evaluation  **2.1.3** **Exclusion criteria**  (1) The exclusion criteria of the children:acute painful disease,critical disease, neurocognitive disorders, communication disorders, use of analgesics,concurrent participation in other studies.  (2)The exclusion criteria for parents of the children: concurrent participation in other studies.  **2.1.4 Termination criteria**  Those who quit the study midway; Patients who develop serious co-morbidities or adverse events during the course of the treatment regimen that make continuation of the trial inadvisable.  **2.1.5 Sample estimation method**  Reference measurement data two-sample mean comparison sample content estimation formula: n_1_=n_2_=2(t_α_+t_β_) ^2^σ^2^/ δ^2^  where δ=μ_1_-μ_2_, is the difference between the means of the two groups, and σ is the mean of the standard deviation of the two groups, based on α=0.05, β=0.1 check the table to get t_α_=1.6449,t_β_=1.2816. The sample of the test group was n_1_, and that of the control group was n_2_. Referring to the relevant literature (12), σ^2^ is 4.73, and δ^2^ is 1.74, and n=47 is obtained by substituting the above data into n_1_=n_2_=2(t_α_+t_β_) ^2^σ^2^/ δ^2^. In addition, the rate of loss to follow-up was calculated to be 10%. As the final sample size was calculated to be 52 cases, 52 cases were taken from each of the two groups.  **2.1.6 Grouping method**  To avoid mutual influence, using the convenience sampling method, children admitted from March 2022 to July 2022 were used as the control group, and children admitted from August 2022 to December 2022 were used as the test group, with 52 cases in each group.  2. **2 Methods of intervention**  Both groups utilized the same model of indwelling needle. Venipuncture was performed by members of the intravenous therapy team using standardized operating procedures.  **2.2.1 Standard intervention**  The control group children received standard care for related diseases after admissionThis included medication care, diet care, life care, disease care, and health education.At the time of venipuncture, we verified the child’s basic information, helped them assume an appropriate position, and informed the child and parents about the purpose of the needle and related precautions. We answered the child's and parent's questions and provided verbal encouragement to the child during venipuncture. In the experimental group, family participatory clown therapy was implemented based on standard care, and the interventions are described below.  **2.2.2 Family participatory clown therap procedurey**  In the test group, family participatory clown therapy was implemented based on conventional care, and the interventions are described below.  (1) Establishing a family participatory clown therapy team  There were seven team members: two for intervention implementation, two for data collection and analysis, and one for coordination and quality control during the intervention; in addition, there was one pediatrician and one nurse to assist the interventionist in implementing the intervention.  (2) Identifying intervention programs  Through reviewing the literature, interviews, and group discussions, referring to the practical experience at home and abroad, and combining the characteristics of children’s psychological development and the actual situation in China, we propose the preliminary development of a localized family participatory clown care program for inpatient children with venipuncture. Pre-experimentation and expert consultation on the preliminary plan will be conducted. The plan will be further improved according to the problems found in the pre-experimentation, as well as the experts’ opinions, to form the final draft of the intervention plan.  (3 )Implementation of interventions  According to the venipuncture procedure, the family participatory clown therapy intervention was conducted in three phases: before, during, and after the venipuncture, with two interveners and one parent of the child. During the intervention, using localized Chinese cartoon images dressed up as comical clowns, the child’s attention was drawn through a series of games (e.g., role-play, games, situational simulation, and improvisation) to reduce the pain and anxiety of the venipuncture experience by diverting the child from nervousness and fear. The details of the specific program are as follows.   | **Intervention time** | **Intervention method** | **Intervention measures** | | --- | --- | --- | | **Before venipuncture**  **（20 minutes）** | Parent training, role-playing, atmosphere creation, improvisation, games, mimicking the piercing process | ①The intervenor begins by communicating with the child and their parents to understand the child's age and hobbies, assess their emotional state and cognitive development, and introduce and train the parents on family participatory clown therapy. This includes explaining the source and purpose of clown care, the process and content of parental involvement, performance skills, and key points of cooperation.The interventionist asked the child about their favorite cartoon character and then dressed in the corresponding costume or held the corresponding doll to communicate with the child. The intervenor performed relevant improvisation based on the child's preferences to establish a trusting relationship.②Intervenor engage children and their parents in various activities such as balloon blowing, clay modeling, painting, origami, puzzles, doll play, storytelling, and magic tricks. Children are given the freedom to choose their preferred dolls, props, and balloon shapes.Through games, we can create a relaxed and joyful atmosphere to reduce the children's unfamiliarity with their surroundings. This will reduce their fear of medical treatment.③The intervenor simulated a medical operation situation with the children and their parents to reduce the children's fear of medical operations.For example,a toy needle or a long balloon can be used as a needle to simulate venipuncture with the child and their parents. This procedure aims to familiarize the child with venipuncture, reducing their fear of needles. Finally, the intervenor guides the parents and child to the venipuncture site using playful techniques such as singing, dancing, and imitating animal movements. | | **During venipuncture**  **(10-15 minutes)** | Stickers, agreements, cartoons, music, dolls, role-play, improvisation, and praise | ①Before disinfecting the skin, the intervenor gently stroked the child's hand, comforted the child, and verbally encouraged the child by placing the child's favorite cartoon sticker on the child's arm or forehead. There is an agreement with the child that if the venipuncture is done well, there will be a mystery reward at the end of the procedure.②During venipuncture, create a comfortable environment for the child. This can be achieved by playing the child's favorite cartoons or music related to the hospital theme. Additionally, allowing the child to hold a favorite doll can provide a sense of security. The intervener and parents communicate with the child during the video storyline, imitating the venipuncture process and providing verbal cues to distract and relieve the child's tension. For example, they may say 'Ultraman can send energy to your body through this tube so that we can defeat the monsters.'During venipuncture, it is important to pay attention to timing and provide encouragement and praise to children to make them feel comfortable. | | **After venipuncture**  **(5-10 minutes)** | Praise, rewards, improvisation, developing protocols, discussing feelings | ①Continue to interact with the child through performing, playing, and storytelling.②The child and parents were escorted to the ward. During this time, the child was commended for their bravery and given a pre-agreed gift as a reward for their behavior during the venipuncture③Develop a protocol with the child for the next venipuncture.④Discuss with the children and parents their feedback on participating in the program and gather suggestions for improvement. |   **2.3 Observed Indicators**  **2.3.1 Pain assessment**  The FLACC scale (13) was used to objectively assess the pain response of the children, including facial expression, crying, body position, limb movement, and comfort degree. Each item was scored as 0, 1, or 2, with a total score of 0–10, where a higher the score indicated a more painful experience . The Wong-Baker Face Pain Scale (W-B FPS) (14) was used to provide a subjective assessment of the pain experienced by the child. The W-B FPS consisted of six facial expressions ranging from smiling to calm to crying on a scale of 0 to 10. Children were asked to indicate which expression best represented their pain level during venipuncture, with higher scores indicating more pain.  **2.3.2 Anxiety assessment of the child**  The Visual Analogue Scale for Anxiety (VAS-A) is a 10-cm-long line, with the leftmost (0 cm) portion being “calm, no anxiety” and the rightmost (10 cm) portion being “very anxious;” the higher the score, the higher the anxiety level (15). As the VAS-A is designed for children six years and older who can understand and complete the task, in this study, the child’s parents reported on his or her behalf, indicating a location on the line to indicate the child’s level of anxiety at that time.  **2.3.3 Medical fear assessment**  The Children’s Fear Scale (CFS) was used to assess the children’s medical fears. The CFS was developed by McMurtry et al. (16) in 2011 to measure the fear of children undergoing a painful medical procedure. A total CFS score of 0 to 4 depicts five faces ranging from a neutral expression (0 = no fear) to a fearful expression (4 = extreme fear). The child is shown these faces and asked to choose the one that shows how scared he or she is at the time.  **2.3.4 Venipuncture crying situation**  The children were observed for crying during venipuncture and one minute after venipuncture  **2.3.5** **Successful rate of one venipuncture**  The one-time success rate of venipuncture was observed.  **2.3.6 Compliance during venipuncture**  Compliance was categorized as three types. Poor compliance indicated children who were strongly resistant and unable to cooperate after intervention, persuasion, and reassurance, requiring assistance with restraint for venipuncture. General compliance indicated children who were mildly resistant but could cooperate after persuasion and reassurance. Good compliance indicated complete and active cooperation without assistance.  **2.3.7 Anxiety assessment of the child’s parents**  The State Anxiety Inventory (S-AI) (17) developed by Spielberger et al. was used to assess anxiety in stressful situations; it was designed to reflect immediate or recent experiences or feelings of fear, tension, apprehension, and nervousness at a specific time.The scale consists of 20 questions and with scores ranging from 1 to 4 (1=not at all, 2=some, 3=moderate, 4=very pronounced; i.e., positive emotions are scored in reverse order), where higher scores indicated a higher anxiety level. The child’s parents selected the appropriate level of anxiety based on their own experience.  **2.3.8 Satisfaction rating of parents of children**   Parental satisfaction was evaluated by asking the following question: “How satisfied are you with the overall performance of venipuncture?” Parental satisfaction was evaluated on a Likert scale of ranging from 1 to 5 (1 = unsatisfied, 2 = relatively unsatisfied, 3 = generally satisfied, 4 = relatively satisfied, 5 = very satisfied). This index was collected 10 min after venipuncture completion (18). 2.4.Quality control (1) Before the start of the formal research trial, a pre-test was conducted to familiarize the researcher and the participating team members with the research process, data collection methods, intervention methods, scale filling methods, etc., and to improve the problems found in the pre-test in a timely manner, so as to ensure that the formal trial is carried out in an orderly manner.  （2）Completion of the intervention by a fixed intervener reduces variation between interviewees and ensures the quality of the intervention.  （3）Data collectors use standardized instructions for questions and surveys to ensure consistency in data collection. Data are collected and reviewed on the spot to ensure validity and completeness.  **2.5. Statistical Analysis Methods**  Statistical analysis was performed on the data using SPSS 21.0 software. The measurement data were presented as mean ± standard deviation. For data meeting parametric assumptions, two-sample independent t-tests were used for between-group comparisons and paired t-tests for within-group comparisons. In the case of non-parametric assumptions, the independent Mann-Whitney test was used for between-group comparisons and the paired Wilcoxon test for within-group comparisons.Count data were expressed as frequencies and percentages, and the Chi-squared test was used to compare between groups.Repeated-measures analysis of variance (ANOVA) was used to analyze the data collected at multiple time points.  **2.6. Ethical principles**   1. Voluntary principle: explain to the parents of the children in detail before the start of the study, sign the informed consent form after obtaining the consent of the parents of the children, the children and their parents voluntarily participate in the study, and have the right to withdraw from the study at any time during the study period without jeopardizing the rights of the other related rights. 2. Principle of confidentiality: All personal information provided by the research subjects during the study shall not be disclosed. |
| --- | --- | --- | --- | --- | --- | --- | --- | --- | --- | --- | --- | --- |
| **3、Technical route of the project study**  Literature review  interview  Research program design  (Selection of study population, inclusion and exclusion criteria, determination of grouping methodology, initial development of study protocol, selection of study instruments)  Eligible study subjects were enrolled and grouped  Control group: 52 cases  Experimental group: 52 cases  Usual treatment and care  Usual treatment and care﹢Family participatory clown therapy  Pain level, anxiety level, compliance, success rate of one venipuncture, crying and 1min stopping rate, children's medical fear, parents' anxiety level and satisfaction were collected from both groups.  Organize data, statistical analysis  panel discussion  Pre-experimentation and expert consultation to refine the study protocol and confirm the final intervention plan  Writing a Manuscript |
| **4.Scheduling of the study**  (1) Preparation stage (October 2021 - March 2022)  A:Literature research, establish the team.  B:Develop preliminary program.  C:Conduct small sample preexperiment, consult with experts, confirm the final program.  (2)Implementation phase (March 2022 - December 2022)  A:Select research subjects who meet the criteria for enrollment, sign the informed consent form, and fill out the baseline questionnaire.  B:Carry out the research experiment and collect data at each corresponding stage.  (3) finalization stage (January 2023 - December 2023)  A:Sort out the data and conduct statistical analysis.  B:Writing a Manuscript. |
