# Supplementary material for: Family participatory clown therapy in venipuncture in hospitalized children: A non-randomized controlled trial
Source: PLoS One. 2024 Jul 25;19(7):e0305101. doi: 10.1371/journal.pone.0305101 (PMC11271897; doi:10.1371/journal.pone.0305101)
Supplement: S1 Fig — (DOC) [file pone.0305101.s004.doc]

**S1 Fig.** **Flow of participants through each phase of the study**

Based on the inclusion and exclusion criteria, we recruited 104 eligible children and their parents.

Control group: 52 cases

Experimental group:52 cases

Standard care

Standard care﹢Family participatory clown therapy法

Data collected from the two groups of children included pain level, anxiety level, compliance, success rate of one venipuncture, crying incidence, rate of stopping crying 1 min after venipuncture, children's medical fear level, parents' anxiety level, and satisfaction.

Basic information, pre-intervention anxiety levels, and children's medical fear levels were collected from children and parents in both groups.

Control group: 52 cases

Experimental group:52 cases
